# Correlation of ADC values of adult brain tumors with the diagnosis and pathological grade: A cross‐sectional multicenter study

**DOI:** 10.1002/hsr2.2110

**Published:** 2024-06-04

**Authors:** Ehsan Hassannejad, Mahtab Mohammadifard, Asma Payandeh, Bita Bijari, Ahmad Shoja, Mostafa Abdollahi, Mahyar Mohammadifard

**Affiliations:** ^1^ Department of Radiology, Faculty of Medicine Birjand University of Medical Sciences Birjand Iran; ^2^ Department of Pathology, Faculty of Medicine Birjand University of Medical Sciences Birjand Iran; ^3^ Faculty of Medicine Mashhad University of Medical Sciences Mashhad Iran; ^4^ Community Medicine department, Faculty of Medicine, Birjand University of Medical Sciences Cardiovascular Diseases Research Center of Birjand University of Medical Sciences Birjand Iran; ^5^ Department of Radiology, School of Medicine, Shahid Modarres Hospital Shahid Beheshti University of Medical Sciences Tehran Iran

**Keywords:** ADC, brain MRI, DWI, tumor

## Abstract

**Background and Aim:**

Brain tumors are common, requiring physicians to have a precise understanding of them for accurate diagnosis and treatment. Considering that various histological tumor types present different cellularity, we conducted this research to examine the role of apparent diffusion coefficient (ADC) values in the differential diagnosis and pathologic grading of brain tumor types.

**Methods:**

In this cross‐sectional study, we gathered pathology reports of histological samples of adult brain tumors. The tissue sample of brain tumors were examined histologically by a pathologist. The magnetic resonance imaging data of these patients were interpreted by a neuroradiologist. The measured ADC values and ADC ratios were calculated. Standard mean ADC values were expressed as 10^−^^6^ mm^2^/s. The findings were compared according to the histological diagnosis of each tumor.

**Results:**

Sixty‐eight patients were included in the study: 34 (50%) were male, and 34 (50%) were female. The average age of the patients was 51.69 + 16.40 years. In the examination of tumor type, 16 (23.5%) were astrocytoma, 9 (13.2%) were oligodendroglioma, 20 (29.4%) were glioblastoma, 4 (5.9%) were medulloblastoma, and 19 (27.9%) were metastatic tumors. the average value of ADC was statistically significantly different according to the pathological type of tumor (*p* < 0.001). The two‐by‐two comparison of average ADC among tumor types revealed significant differences, except for oligodendroglioma and glioblastoma (*p*‐value = 0.87) and glioblastoma and medulloblastoma (*p*‐value = 0.347). The average value of ADC and ADC ratio was statistically significantly different according to the pathological grade of the tumor (*p* < 0.001). In the two‐by‐two comparison of average ADC between all pathological grades of the tumor showed a significance difference except for Grade I and Grade II (*p*‐value = 0.355). The mean value of ADC and ADC ratio for glioblastoma and metastatic tumors showed no significant difference.

**Conclusion:**

The assessment of brain tumor grade through ADC examination will help to estimate prognosis and devising suitable therapeutic strategies.

## 1 INTRODUCTION

Brain tumors are common, requiring physicians to have a precise understanding of them for accurate diagnosis and treatment. The worldwide incidence of central nervous system (CNS) neoplasms is estimated to be around 771,110 cases over 5 years. The incidence varies depending on age, gender, race, and region. CNS neoplasms contribute to 2.71% of cancer‐related deaths globally. Mortality rates are higher in the adult population compared to children. The most common brain tumors include intracranial metastasis, meningiomas, and gliomas. It is noteworthy that the occurrence of brain tumors is increasing, possibly because of progress in primary brain tumor diagnosis or advances in treatment methods and survival outcomes for patients with systemic cancers.^1^, ^2^, ^3^

Magnetic resonance imaging (MRI) provides good information about the type and grade of tumors. Diffusion‐weighted imaging (DWI) sequences in MRI provide additional data derived from the microscopic movement of water molecules that are unattainable through conventional MRI. DWI has been used in various studies to evaluate the tumor grade or tumor type differentiation and to diagnose other brain space‐occupying lesions.

DWI sequences are limited in that they not only depict the state of microscopic movements of water molecules but also show information about blood flow and tissue characteristics associated with T2 relaxation time. Thus, its images do not faithfully reflect the pure state of water molecule movements, creating challenges in quantitative evaluation. Calculating the apparent diffusion coefficient (ADC) values makes it possible to evaluate the net diffusion of water molecules while eliminating superfluous information.^4^, ^5^

The diffusion of water molecules within tissue is reliant on various factors. One factor that restricts the diffusion of water molecules is the density of cells within the tissue and the nuclear‐to‐cytoplasmic volume ratio, which usually increases in high‐grade tumors.^6^, ^7^, ^8^

Considering that various histological tumor types present different cellularity, we conducted this research to examine the role of ADC values in the differential diagnosis and pathologic grading of brain tumor types.

## 2 METHOD

Our current study was designed as a cross‐sectional investigation. We gathered pathology reports of histological samples of adult brain tumors from Birjand Imam Reza, Valiasr, and Razi hospitals. A pathologist examined the samples following the World Health Organization (WHO)−2016 classification. Tumors categorized as WHO grade I and II were classified as low grade, whereas tumors classified as grade III and IV were categorized as high grade. The samples collected from medical centers during the period of 2021–2023 were included in the study.

Patients who did not have diagnostic pathological findings for the sampled tumor type, acceptable pathology reports, adequate quality MRI, or did not undergo DWI and ADC sequences were excluded from the study.

Also, the areas of bleeding, necrosis, cystic changes, or severe calcification that lead to disturbance in measuring ADC values were removed from the samples. The identification of tumor locations and characterizations in these patients was done through MRI scans, both with and without contrast injection. The acquisition of ADC and DWI images was performed using single‐shot spin‐echo sequences on an MRI system operating at a magnetic field strength of 1.5 Tesla. All MRIs were obtained before any surgical intervention or manipulation, such as biopsy. The MRI information was obtained from the PACS system based on the patient's medical record numbers. Conventional MRI techniques were performed, yielding images with an average slice thickness of 5 mm, including axial T1‐weighted images, axial T2‐weighted images, axial fluid‐attenuated inversion recovery images, and sagittal T1‐weighted images. A post‐contrast MRI was carried out after intravenous injection of a Gadolinium‐based contrast agent (Gd‐DTPA) at a dose of 0.1–0.2 mL/kg. To identify the tumor's location and its components, as well as determine the area for measuring ADC values, post‐contrast T1‐weighted images were gained in axial, sagittal, and coronal planes.

The ADC and DWI images, obtained with *b* values of 0 and 1000 s/mm^2^, were evaluated for cellularity and tumor density by a neuroradiologist who was blinded to the histopathological results.

**Figure 1:**
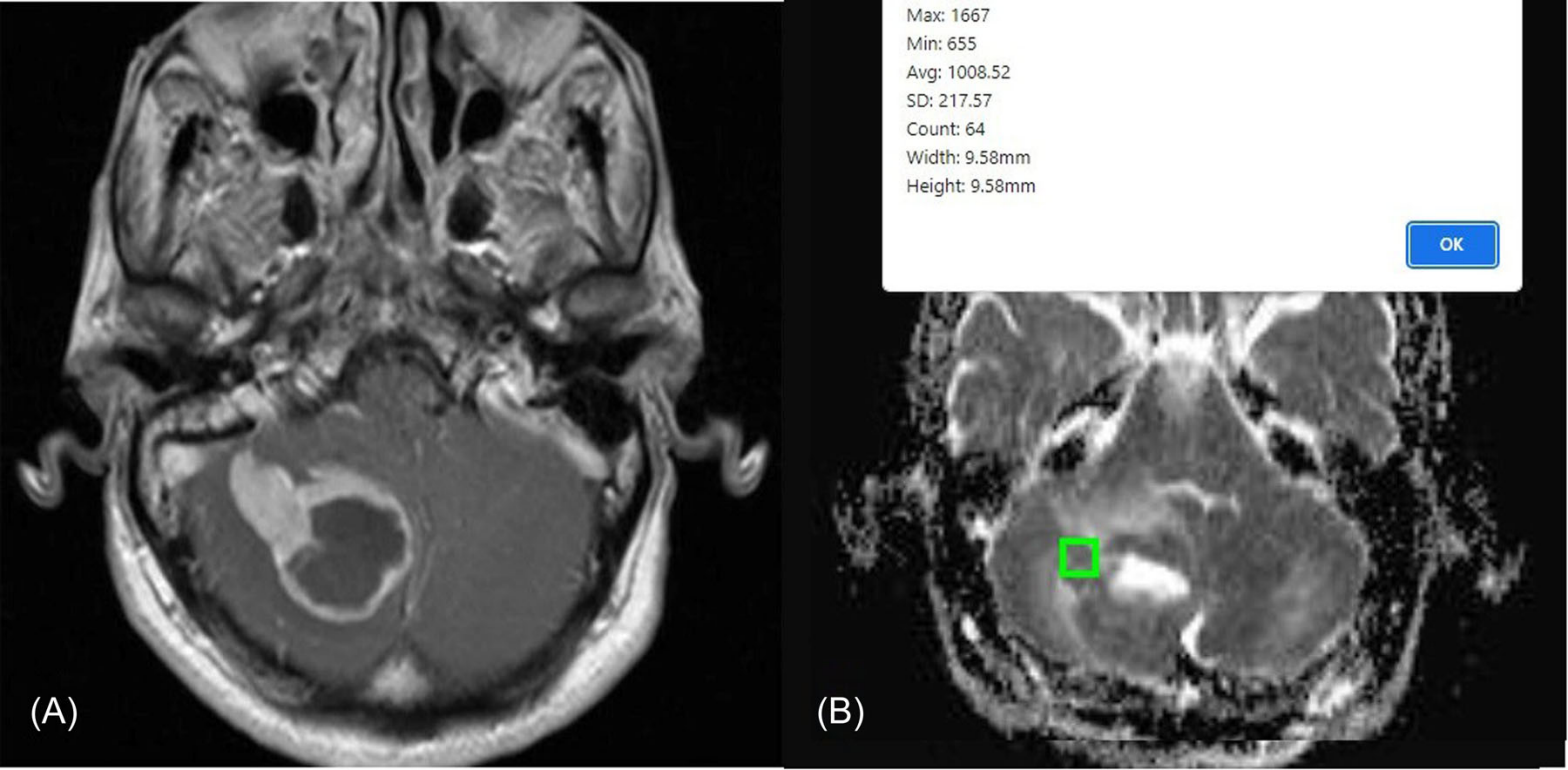
(A) Contrast‐enhanced axial T1‐weighted MRI of a brain tumor for identifying the tumor's location and its components. (B) measuring the apparent diffusion coefficient (ADC) value by placing region of interest (ROI) on the darkest point of the solid section of the tumor in the ADC sequence.

To measure the ADC value, a region of interest was situated on the darkest point of the solid section of the tumor in the ADC sequence. Areas of edema, hemorrhage, or necrosis were excluded from the measurements. An area of approximately 1 square centimeter was measured in three consecutive sections, and the average value was subsequently reported (Figure 1). Standard mean ADC values were expressed as 10^−6^ mm^2^/s.

For the purpose of improving the accuracy and reproducibility of the study, the ADC ratio was also determined. The ADC ratio was obtained by dividing the average ADC of the tumor by the ADC of the normal contralateral white matter at the same cross‐sectional level.

The ADC values and ADC ratios for each tumor pathological type were obtained and subsequently analyzed.

The data was entered into statistical package for social sciences (SPSS) software version 22.0, and centrality and dispersion indices (mean, standard deviation), frequency distribution, and percentage were used to report descriptive data.

The Kolmogorov–Smirnov test was conducted to assess the normality of data distribution. If the distribution was normal, statistical tests of analysis of variance and Tukey's post hoc test were performed. If the distribution was non‐normal, statistical tests of Kruskalvallis and Mann–Whitney‐*U* were conducted at a significant level of *p*‐value = 0.05. All statistical tests were applied two‐sided.

## 3 ETHICAL CONSIDERATIONS

The information of all patients was kept confidential with the project manager. All methods were implemented in compliance with the ethical regulations mandated by the research committees of the University of Medical Sciences or the Declaration of Helsinki. The project was carried out after approval by the Research Council of the Faculty of Medicine, IR.BUMS.REC.1402.279. All patients were informed and signed the informed consent and to maintain confidentiality, all data were entered into the researcher's checklist by coding.

## 4 RESULTS

Sixty‐eight patients were included in the study: 34 (50%) were male and 34 (50%) were female. The average age of the patients was 51.69 + 16.40 years. In the examination of tumor type, 16 (23.5%) were astrocytoma, 9 (13.2%) were oligodendroglioma, 20 (29.4%) were glioblastoma, 4 (5.9%) were medulloblastoma, and 19 (27.9%) were metastatic tumors.

Also, in examining the grades of tumors, 2 (4.1%) were grade I, 12 (24.5%) were grade II, 11 (22.4%) were grade III, and 24 (49%) were grade IV. Also, 14 (28.6%) and 35 (71.4%) tumors were low and high‐grade, respectively.

The average ADC and ADC ratio in all cases was 1080.96 ± 214.63 and 1.34 ± 0.26, respectively.

Table 1 compares the average ADC value and ADC ratio according to the tumor type and pathological diagnosis. As seen, the average value of ADC was statistically significantly different according to the pathological type of tumor.

**Table 1:** Comparison of the average ADC value and ADC ratio according to the tumor type and pathological diagnosis.

Tumor type	Average ADC value	*p*‐Value	ADC ratio	*p*‐Value
Astrocytoma	1335.25 ± 215.92	<0.001	1.61 ± 0.27	<0.001
Oligodendroglioma	1131.22 ± 167.93		1.46 ± 0.23	
Glioblastoma	976.85 ± 101.64		1.26 ± 0.14	
Medulloblastoma	831.00 ± 65.07		1.12 ± 0.09	

Abbreviation: ADC, apparent diffusion coefficient.

The two‐by‐two comparison of average ADC among tumor types revealed significant differences in Tukey's post hoc test, except for oligodendroglioma and glioblastoma (*p*‐value = 0.087) and also between glioblastoma and medulloblastoma (*p*‐value = 0.347).

Table 2 compares the average ADC value and ADC ratio according to the pathological grade of the tumor. As seen, the average value of ADC and ADC ratio was statistically significantly different according to the pathological grade of the tumor.

**Table 2:** Comparison of the average ADC value and ADC ratio according to the pathological grade of the tumor.

Tumor grade	Average ADC value	*p*‐Value	ADC ratio	*p*‐Value
Grade I	1544.50 ± 81.31	<0.001	2.05 ± 0.09	<0.001
Grade II	1377.92 ± 173.97		1.68 ± 0.19	
Grade III	1083.73 ± 124.40		1.33 ± 0.06	
Grade IV	952.54 ± 110.31		1.23 ± 0.14	

Abbreviation: ADC, apparent diffusion coefficient.

In the two‐by‐two comparison of average ADC between all pathological grades of the tumor, Tukey's post hoc test showed significant difference except for Grade I and Grade II (*p*‐value = 0.355).

Table 3 shows the comparison of the average value of ADC and ADC ratio according to the low and high tumor grade. The average value of ADC and ADC ratio was statistically significantly different according to the low and high tumor grade.

**Table 3:** Comparison of the average ADC value and ADC ratio according to the low and high tumor grade.

Tumor grade	Average ADC value	*p*‐Value	ADC ratio	*p*‐Value
Low grade	1401.71 ± 172.56	<0.001	1.73 ± 0.22	<0.001
High grade	993.77 ± 128.85		1.26 ± 0.13	

Abbreviation: ADC, apparent diffusion coefficient.

**Figure 2:**
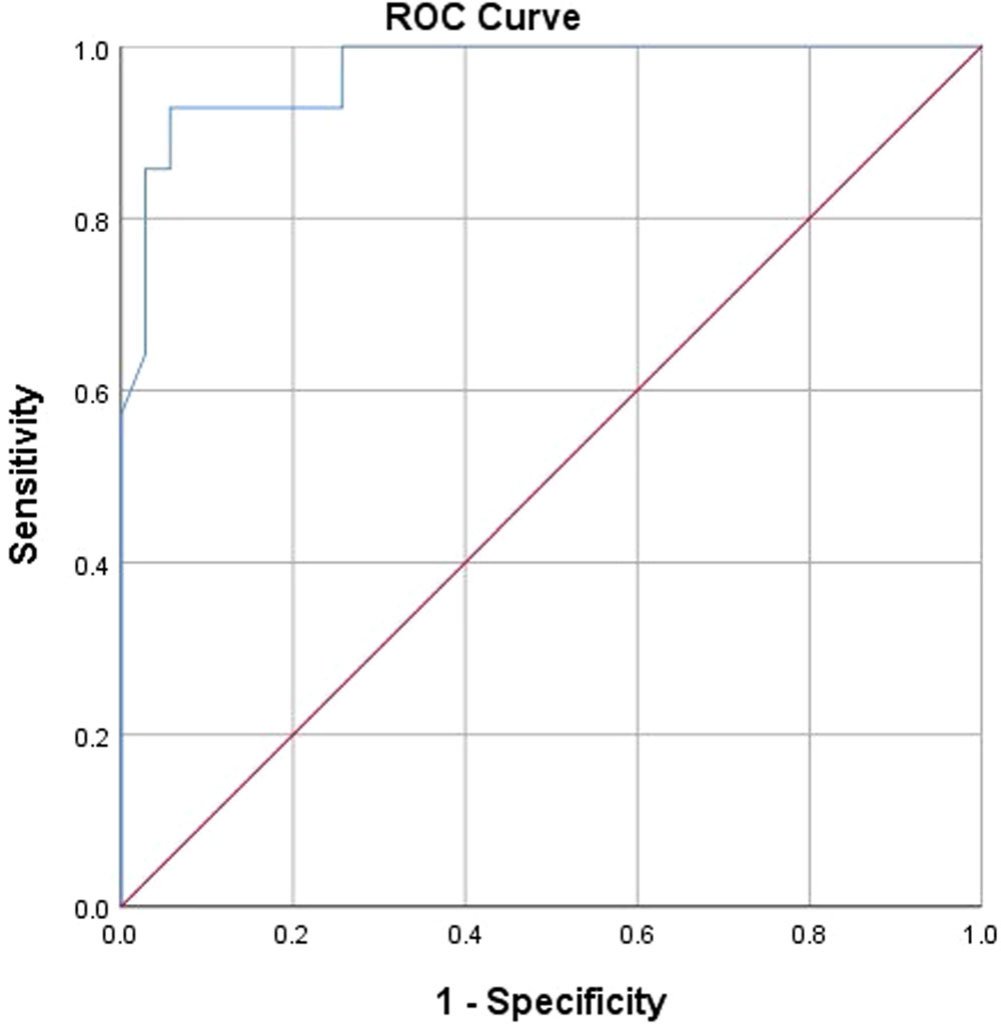
ROC curve for the mean ADC differentiating high‐grade tumors from low grade ones. AD, apparent diffusion coefficient.

Figure 2 shows the ROC curve for the mean ADC, differentiating high‐grade tumors from low‐grade ones. The mean ADC value that can differentiate high‐grade tumors from benign ones was calculated by the ROC curve to be 1055 (sensitivity of 100% and specificity of 74.3%, and with area under the curve of 0.970), and a brain tumor with a mean ADC value equal to or lower than this value is most probably high grade.

**Figure 3:**
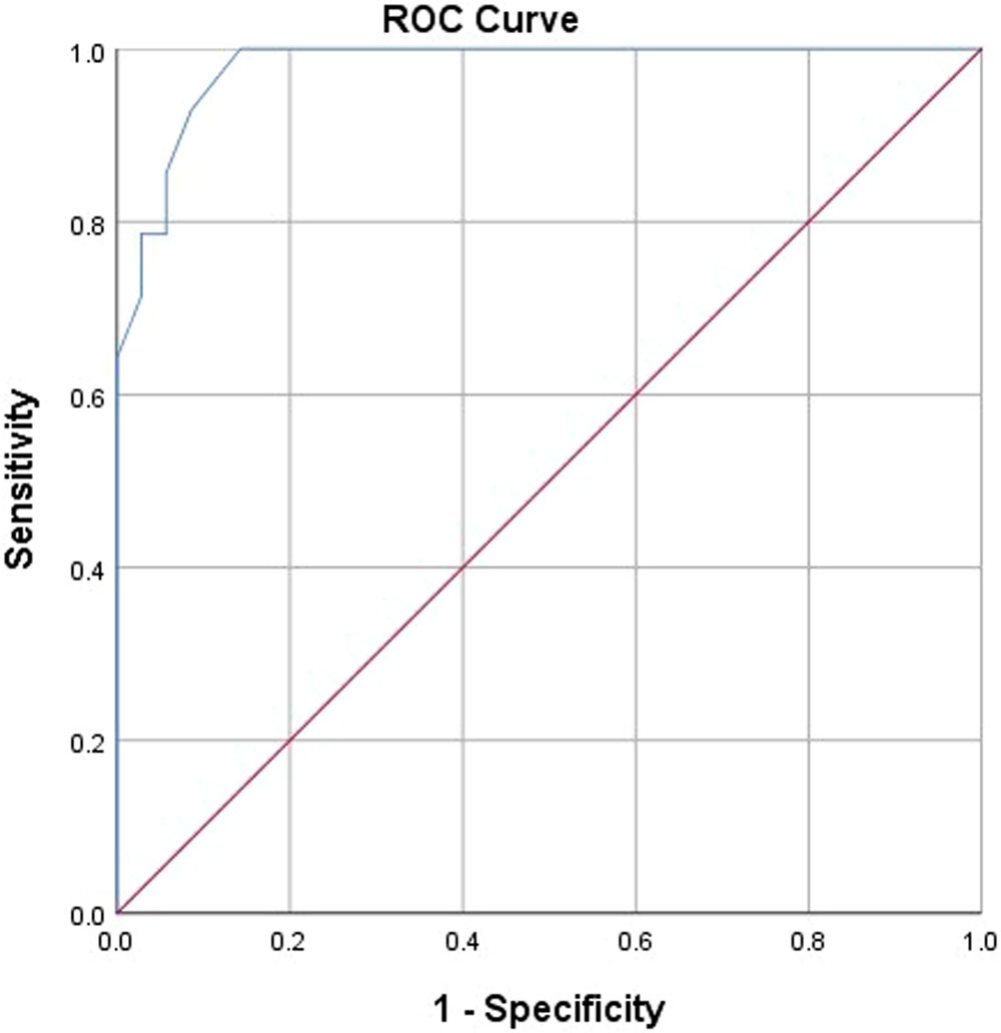
ROC curve for the ADC ratio differentiating high‐grade tumors from low‐grade ones. ADC, apparent diffusion coefficient.

Figure 3 shows the ROC curve for the ADC ratio differentiating high‐grade tumors from low‐grade ones. The ADC ratio that can differentiate high‐grade tumors from benign ones was calculated by the ROC curve to be 1.40 (sensitivity of 100% and specificity of 85.7%, and with area under the curve of 0.980), and a brain tumor with an ADC ratio equal to or lower than this value is most probably high grade.

Table 4 compares the average value of ADC and ADC ratio of glioblastoma and metastatic tumors.

**Table 4:** Comparison of the average ADC value and ADC ratio of glioblastoma and metastatic tumors.

	Average ADC value	*p*‐Value	ADC ratio	*p*‐Value
Glioblastoma	976.85 ± 101.64	0.462	1.26 ± 0.14	0.147
Metastatic tumor	1005.21 ± 133.20		1.19 ± 0.14	

Abbreviation: ADC, apparent diffusion coefficient.

The mean value of ADC and ADC ratio for glioblastoma and metastatic tumors showed no significant difference.

## 5 DISCUSSION

The preferred neuroimaging technique for detecting and evaluating brain tumors is MRI. It is because of its exceptional soft tissue differentiation capabilities, precise delineation of anatomic localization for surgical planning, and the ability to assess tumor response to therapy.^9^ Nevertheless, it does not offer information on cellularity or the extent of the lesion and provides restricted information concerning the type and grade of the tumor.^10^, ^11^ Thus, incorporating further imaging modalities like DWI can assist in distinguishing brain tumor types. The inspection of tumor tissue characteristics can be accomplished through DWI, an MRI technique based on water molecules' random microscopic (Brownian) motion. The analysis of ADC map images enables the examination of water molecule diffusion characteristics in a manner that is independent of the T2 characteristics of the tissue observed in the DWI sequence.^12^ The increased cellular density of high‐grade tumors limits the microscopic water movement in the tissue, leading to lower ADC values compared to low‐grade tumors.^13^

Various determinants influence the rate of apparent diffusion in brain tissues, primarily including the degree of tumoral cellularity, the extent of neuroarchitectural destruction, the size of extracellular space pores, and the presence of vasogenic edema.^14^, ^15^, ^16^ The calculated ADC values are determined by diffusion in the extracellular space, rather than the diffusion of water molecules within every single cell, which has a negligible impact on the overall value.^17^

In our study, we investigated the role of ADC values of adult brain tumors and the ratio of ADC values of tumors to normal brain parenchyma in the grading of brain tumors before surgery.

The present study highlights noteworthy differences in the ADC value and ADC ratio among various histological groups of adult brain tumors, including astrocytoma, oligodendroglioma, glioblastoma, and medulloblastoma. The ADC values and ADC ratios could discriminate between the high‐grade and low‐grade groups of tumors. Our findings suggest a correlation between lower ADCs and high‐grade tumors, while higher ADCs and ADC ratios are indicative of low‐grade tumors. These findings can be aptly clarified by considering the information obtained from previous studies.

In Yamasaki's retrospective study, which investigated ADC in brain tumors, 275 patients with an average age of 45.9 + 21.5 were studied. Only the solid tumor components were chosen as regions of interest in this study. The cystic, necrotic, and hemorrhagic tumor areas were excluded. A significant inverse relationship was observed between ADC and astrocytic tumors of WHO grades II–IV (grade II vs. grades III and IV (*p* < 0.01); grade III vs. IV (*p* < 0.01). A significant difference was noted between low‐grade (grades I and II) and high‐grade (grades III and IV) glioneuronal tumors (*p* < 0.01). Similar to our study, glioblastomas and metastatic tumors could not be discriminated based on ADC values. Unlike our article, that study could not discriminate between astrocytic and oligodendroglial tumors. However, this difference can be due to the fact that Yamasaki's study contained only one oligodendroglial tumor.^18^

The study conducted by Kitis aimed to determine the potential of DWI using minimum apparent diffusion coefficient (ADCmin) values to differentiate various types of brain tumors. The study, which involved 65 patients, it was shown that, similar to our own study, the ADCmin values of low‐grade gliomas (1.09 ± 0.20 × 10^−3^ mm^2^/s) were significantly higher (*p* < 0.001) than those of other high‐grade gliomas. (*p* < 0.01). Moreover, no statistically significant differences were found between glioblastomas (0.70 ± 0.16 × 10^−3^ mm^2^/s) and metastases (0.78 ± 0.21 × 10^−3^ mm^2^/s).^19^

Similar results were obtained in the Kono study. Within the context of the study, it was observed that patients diagnosed with glioblastoma exhibited lower ADC values compared to those with grade II astrocytoma (*p* = 0008). The ADC did not differ significantly between patients with glioblastomas and those with metastatic tumors.^20^

Contrary to our study and the studies mentioned, it has been shown in other studies that ADC can distinguish a metastasis from glioblastoma.

In the study performed by Chiang, the tumoral ADC values of high‐grade gliomas and metastases were observed to be 1.04 ± 0.42 × 10^−3^ mm^2^/s and 1.87 ± 0.73 × 10^−3^ mm^2^/s, respectively. A significant difference between the two was noted.^21^

In Krabe's study, it was observed that cerebral metastases exhibited a considerably higher ADC than high‐grade gliomas, with a significance level of *p* < 0.05.^22^

The Server study demonstrated a significant difference in the mean ADC level between high‐grade glioma and metastases, with values of 0.986 + 0.274 × 10^−3^ mm^2^/s and 0.819 + 0.228 × 10^−3^ mm^2^/s, respectively (*p* < 0.05).^23^

When explaining this distinction, it is worth mentioning that high‐grade gliomas may have higher ADC values as a result of microcystic degeneration, foci of necrosis, and the excessive production of extracellular matrix components by glioblastoma cells.^24^, ^25^ However, brain metastases represent a heterogeneous and varied group of tumors, and their microscopic characteristics may be influenced by their origin. This matter has the potential to yield varying outcomes across different studies. Furthermore, there are limitations to some studies, including a small sample size.

The differentiation of metastasis from other malignant tumors is not a diagnostic dilemma when the patient has primary cancer and presents with multiple lesions. Nonetheless, the differentiation between solitary metastasis and glioblastoma can pose difficulties in the absence of a known primary cancer. Therefore, due to the importance of this issue, to further investigate the role of ADC in differentiating between high‐grade glioma and metastases, more extensive studies with a larger number of samples should be conducted.

In our study, ADC values could not differentiate between oligodendroglioma and glioblastoma, glioblastoma and medulloblastoma, as well as WHO grade I and II tumors. This issue can be attributed to the fact that the cellularity values of these tumors may be close to each other. Moreover, due to the limited sample size in our study, it is necessary to investigate this issue further by using a larger sample size in future studies.

The ADC values are influenced by the diffusion restriction of water molecules, which is inversely related to the grade and cell density of the tumor. A higher grade and cell density result in a more pronounced restriction of water diffusion, causing the ADC values to decrease, commonly known as diffusion restriction. Tumors classified as WHO grade IV, such as medulloblastoma and glioblastoma, exhibit the lowest ADC values and ADC ratios because of their high cellularity. In contrast, low‐grade tumors such as pilocytic astrocytoma with the lowest cell density show higher ADC values compared to other tumors.

DWI stands out as the most convenient and time‐efficient noninvasive technique for preoperative grading and therapy planning of brain tumors. By employing a multislice, single‐shot echoplanar imaging sequence, which effectively suppresses motion artifacts, one can achieve high‐quality images with high spatial resolution. Single‐shot spin echo echoplanar imaging is fast and relatively resistant to rigid body motion due to its ability to eliminate macroscopic motion. This method is particularly well‐suited for evaluating restless patients because of its advantages in both speed and low motion sensitivity. Moreover, it facilitates the assessment of ADC values in different tumor types, reflecting the restricted diffusion of protons caused by alterations in the structure and cellularity of tumoral tissue.^14^, ^20^, ^26^, ^27^, ^28^

As the molecular markers were not available in some cases, we had to investigate the tumors according to the WHO 2016 classification; however, it is our recommendation that in the years to come, further investigations be carried out to investigate the brain tumors grade through ADC examination according to the newest WHO 2021 classification of CNS tumors including molecular markers.

In conclusion, the assessment of brain tumor grade through ADC examination will help to estimate prognosis and devising suitable therapeutic strategies.

## AUTHOR CONTRIBUTIONS

**Ehsan Hassannejad**: Writing—original draft; writing—review & editing; data curation; methodology; formal analysis; project administration. **Mahtab Mohammadifard**: Data curation; writing—review & editing; visualization. **Asma Payandeh**: Data curation; writing—review & editing; writing—original draft; methodology; formal analysis. **Bita Bijari**: Formal analysis; methodology. **Ahmad Shoja**: Writing—review & editing. **Mostafa Abdollahi**: Writing—review & editing. **Mahyar Mohammadifard**: Conceptualization; investigation; writing—review & editing; methodology; supervision; validation; visualization; writing—original draft.

## CONFLICT OF INTEREST STATEMENT

The authors declare no conflicts of interest.

## ETHICS STATEMENT

The method was approved in compliance with scientific and ethical standards. All methods were performed in line with the relevant guidelines and regulations. The Medical Ethics Committee of Mashhad University of Medical Science approved this study. All patients were informed and signed the informed consent.

## TRANSPARENCY STATEMENT

All authors have read and approved the final version of the manuscript. Mahyar Mohammadifard had full access to all of the data in this study and takes complete responsibility for the integrity of the data and the accuracy of the data analysis. The lead author, Mahyar Mohammadifard, affirms that this manuscript is an honest, accurate, and transparent account of the study being reported, that no important aspects of the study have been omitted, and that any discrepancies from the study as planned (and, if relevant, registered) have been explained.

## Data Availability

The data that support the findings of this study are available on request from the corresponding author. The datasets created during the current study are not publicly accessible due to the possibility of compromising the privacy of individuals.
